# Therapeutic Approaches Targeting Proteins in Tumor-Associated Macrophages and Their Applications in Cancers

**DOI:** 10.3390/biom12030392

**Published:** 2022-03-02

**Authors:** Deyang Wu, Xiaowei Liu, Jingtian Mu, Jin Yang, Fanglong Wu, Hongmei Zhou

**Affiliations:** 1 State Key Laboratory of Oral Diseases, National Center of Stomatology, National Clinical Research Center for Oral Diseases, Frontier Innovation Center for Dental Medicine Plus, Department of Oral Medicine, West China Hospital of Stomatology, Sichuan University, Chengdu 610041, China; scu_wudeyang@163.com (D.W.); jingtianmou@163.com (J.M.); yangjin@scu.edu.cn (J.Y.); 2 State Key Laboratory of Oral Diseases, West China College of Stomatology, Sichuan University, Chengdu 610041, China; 2019151640145@stu.scu.edu.cn

**Keywords:** tumor-associated macrophages, tumor microenvironment, targeted therapy, protein

## Abstract

Tumor-associated macrophages (TAMs) promote tumor proliferation, invasion, angiogenesis, stemness, therapeutic resistance, and immune tolerance in a protein-dependent manner. Therefore, the traditional target paradigms are often insufficient to exterminate tumor cells. These pro-tumoral functions are mediated by the subsets of macrophages that exhibit canonical protein markers, while simultaneously having unique transcriptional features, which makes the proteins expressed on TAMs promising targets during anti-tumor therapy. Herein, TAM-associated protein-dependent target strategies were developed with the aim of either reducing the numbers of TAMs or inhibiting the pro-tumoral functions of TAMs. Furthermore, the recent advances in TAMs associated with tumor metabolism and immunity were extensively exploited to repolarize these TAMs to become anti-tumor elements and reverse the immunosuppressive tumor microenvironment. In this review, we systematically summarize these current studies to fully illustrate the TAM-associated protein targets and their inhibitors, and we highlight the potential clinical applications of targeting the crosstalk among TAMs, tumor cells, and immune cells in anti-tumor therapy.

## 1. Introduction

Tumor-associated macrophages (TAMs), as an integral cellular component in the tumor microenvironment (TME), promote the process involved in tumor progression, including proliferation, infiltration, angiogenesis, metastasis, stemness, immune escape, and therapeutic resistance [1]. Previous studies have shown that TAMs can attenuate therapeutic effects by expressing various pro-tumor cytokines and chemokines, etc., decreasing T-cell infiltration, suppressing the function of immune cells, and fueling tumor cells, respectively [1,2,3]. Blocking the positive effects of TAMs on tumors and/or reducing the number of TAMs in the TME could suppress the tumor-promoting biological behaviors involved in carcinogenesis, progression, invasion, recurrence, and metastasis. For instance, TAMs would be beneficial for tissue remodeling and the construction of the tumor’s physical barrier, which inhibit the infiltration of immune cells, such as CD8^+^ T cells [4].

Since the receptors in TAMs are less likely to undergo mutation, targeting their receptors would be a promising therapeutic approach for anti-tumor therapy. To support this notion, recently, in preclinical models, several special antibodies targeting their receptors were used against TAMs, including ch14.18, duvelisib (IPI-145), and ipilimumab, which were developed in neuroblastoma, T-cell lymphoma, and melanoma, respectively [5,6,7]. However, TAMs are highly plastic stromal cells [8], indicating that any therapeutic strategies developed to exploit the targeting proteins in TAMs should account for their heterogeneous nature in order to optimize treatment efficacy.

Infiltrated and polarized TAMs promote tumor progression in a direct manner through secreting multiple cytokines and/or chemokines, and an indirect manner, through recruiting the surrounding immune cells or remodeling the extracellular matrix (ECM). For instance, in a mouse model, Shono et al. provided evidence showing that Celecoxib inhibited the expression of the C–C motif chemokine ligand 2 (CCL2) and C–X–C motif chemokine receptor 3 (CXCR3) to reduce the recruitment of TAMs [9]. In another study, Chow et al. showed that T-cell immunoglobulin and mucin domain-containing protein 4 (Tim4^+^) macrophages (Tim4, a receptor for phosphatidylserine) combined directly with CD8^+^ T cells to attenuate the anti-tumor immune response [10]. Although these findings are promising, the many obstacles of targeting proteins in TAMs need to be further explored. For instance, the avenue for reducing the earliest recruitment of TAMs might not affect those TAMs that have finished recruitment. Additionally, drug delivery methods for targeting proteins in TAMs are constrained by vascular permeability, the ECM, and the tissue osmotic pressure, for instance, which decrease drug affinity. Indeed, compared with micromolecules, such as calcium ions, proteins, as the executors in many biochemical procedures, are easy to collect and analyze when targeted by drugs. Notably, the three-dimensional structures, intracellular locations, and phosphorylation states of proteins in TAMs can affect the targeting efficacy. Thus, in this review, we systematically summarize the therapeutic approaches for targeting proteins in TAMs with regard to recruitment, polarization, crosstalk with tumor cells, and immune responses, highlighting potential strategies for targeting proteins in TAMs for anti-cancer treatment.

## 2. Targeting Proteins in the Recruitment and Polarization of TAMs

**Figure 1:**
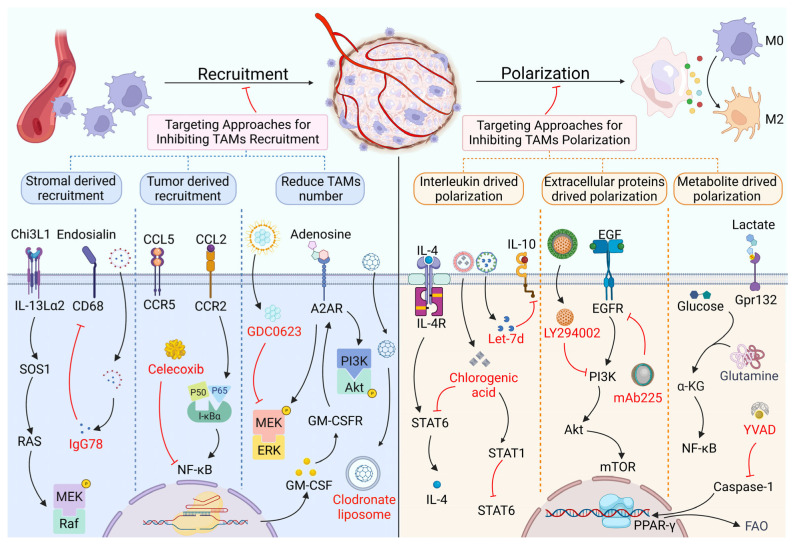
Targeting approaches for inhibiting TAM recruitment and polarization. TAMs are mainly recruited by the factors derived from stromal and tumor cells. These factors, including endosialin, chitinase 3-like protein (Chi3L1), C–C motif chemokine ligand 2 (CCL2), etc., could bind with their receptors in TAMs, then activating the downstream effectors to regulate the recruitment of TAMs through nuclear factor kappa-light-chain-enhancer of activated B cells (NF-κB), phosphoinositide 3-kinase/protein kinase B (PI3K/Akt), mitogen-activated protein kinase (MAPK), etc. signaling pathways. Targeting inhibitors, including IgG78, celecoxib and GDC0623 block CD68, the NF-κB and MEK signaling pathways inhibit TAM recruitment or reduce the number of TAMs. The polarization of TAMs is mediated by interleukins, extracellular proteins and metabolite. Tumor-derived Interleukin 4 (IL-4), IL-10, lactate and endothelial growth factor (EGF) could combine with IL4 receptor, IL-10 receptor, G-protein-coupled receptor 132 (Gpr132), and EGFR on TAMs to induce the polarization of TAMs via downstream signals, including PI3K/Akt/mTOR and signal transducers and activators of transcription 6 (STAT6) cascade. The chlorogenic acid, Let-7d, LY294002, mAb225 and YVAD could inhibit STAT6, IL-10 receptor, PI3K, EGFR and caspase-1 to attenuate TAM polarization.

To date, overwhelming evidence suggests that the main cellular source of TAMs is recruitment and polarization [11,12]. Typically, monocytes or myeloid cells infiltrate from the blood circulation and/or local tissue, and then differentiate into macrophages. Induced by chemokines, cytokines and vascular endothelial growth factor (VEGF), for instance, in the TME, TAMs polarize toward the M1 phenotype (with pro-inflammatory and anti-tumor effects) and M2 phenotype (with anti-inflammatory and pro-tumor functions) to affect tumor progression [13,14]. On the other hand, TAMs can also be directly recruited from the surrounding tissue. Thus, to block TAMs’ pro-tumor efficacy, decreasing their recruitment and attenuating their polarization have been extensively studied (Figure 1).

### 2.1. TAM Recruitment and Its Targeted Therapy Based on Proteins

TAM recruitment is mainly mediated by tumor cells and surrounding stromal cells through the release of diverse chemokines, including CCL5 and CCL2, and stromal glycoproteins can promote tumor progression by facilitating TAM recruitment from the circulation and/or local tissue to the tumor site (Table 1). For instance, Nie et al. showed that CCL5 derived from breast phyllode tumors was involved in TAM recruitment by binding with CCR5 on the membranes of TAMs, and the CCL5 inhibitor, maraviroc, was shown to attenuate TAM recruitment and suppress malignant progression [15], indicating that targeting CCR5 in TAMs might represent a potential strategy for decreasing recruitment in malignant breast phyllode tumors. Furthermore, several studies have shown that the expression of CCL2, followed by TAM recruitment, is positively correlated with the activation of NF-κB [16,17], suggesting that targeting the NF-κB/CCL2 signal might be beneficial for blocking TAM recruitment. In agreement with this, in a mouse model, Shono et al. used celecoxib to suppress NF-κB and found that the downregulation of CCL2 attenuated TAM recruitment and increased the apoptosis of tumor cells in malignant glioma [9]. Collectively, these findings demonstrate that CCL2 might be a key regulator in chemokine-induced TAM recruitment and might exhibit great potential to be targeted in decreasing TAM recruitment.

In stromal cells, we found that the expression of IL-1β in periodontal inflammation tissue was positively correlated with the production of both CCL2 and CCL5 in BC, and performing anakinra, an IL-1β inhibitor, could reduce myeloid-derived suppressor cell (MDSC) recruitment [27]. Yang et al. found that endosialin expressed by cancer-associated fibroblasts (CAFs) can interact with the glycoproteins existing on the surfaces of TAM membranes, such as CD68 (the major biomarker in TAMs), inducing both the recruitment and polarization of TAMs, and this process could be inhibited by IgG78 in hepatocellular carcinoma (HCC) [42], suggesting that endosialin and CD68 might be targeted for TAMs recruitment. However, there are few studies on targeting CD68 in TAM recruitment and polarization. An alternative hypothesis is that CD68 is a kind of lysosomal/endosomal-associated membrane glycoprotein with a smaller fraction on the cell surface, and an anti-CD68 antibody can recognize the antigen, but not trigger the lysosomal/endosomal cascades for functional properties. Another kind of secreted glycoprotein, chitinase 3-like protein (Chi3L1), can also favor the recruitment of TAMs in cancers. To identify the stromal-derived Chi3L1 in TAM infiltration, Cohen et al. found that CAFs-derived Chi3L1 could induce pro-inflammatory signaling in tumor cells, promoting the release of chemokines, such as CCL2, facilitating TAM recruitment and promoting tumor growth in BC [43]. In sum, the above evidence shows that glycoproteins, especially Chi3L1 in stromal cells, including CAFs and TAMs, have the potential to be commonly targeted for blocking TAM recruitment.

Another strategy for decreasing TAM recruitment is to reduce their number directly by the application of inhibitors, clodronate liposomes, among others. On the one hand, since the granulocyte-macrophage colony-stimulating factor (GM-CSF) can upregulate macrophages proliferation [44], Wang et al. showed that TAMs can autocrine GM-CSF and recognize adenosine by the upregulation of A2A receptors, subsequently activating PI3K/Akt and MEK/ERK cascades, increasing the proliferation of macrophages [31]. On the other hand, given that the activity of the p38 MAPK cascade of M1 macrophages induces these cells to be independent of the MEK/ERK pathway, Baumann et al. performed a MEK inhibitor (MEKi), GDC-0623, to block the MEK/ERK pathway and found that M2 macrophages were highly fragile to the MEKi, while the existence of the p38 MAPK cascade can protect M1 macrophages from death induced by MEKi [32]. In our preliminary study, we found that clodronate liposome could significantly reduce TAMs and splenic macrophages, resulting in reduced squamous cell carcinoma volumes [45]. Mechanistically, clodronate liposome can be engulfed by TAMs via phagocytosis, and accumulated clodronate can be released by lysosomal/endosomal systems; therefore, TAMs would be eliminated at a certain intracellular concentration of clodronate [46]. In summary, these studies demonstrate that suppressing TAM proliferation or promoting TAM ablation can effectively reduce the number of TAMs in the TME, attenuating tumor progression.

### 2.2. TAM Polarization and Its Targeted Therapy Based on Proteins

Given that proteins, such as interleukins, can promote the polarization of TAMs toward the M2 phenotype, targeting those interleukins might achieve good anti-tumor efficacy. For instance, Su et al. found that blocking IL-10 by Let-7d was able to inhibit M2 polarization in renal cell carcinoma [47], and Rahal et al. found that exogenous IL-4 and IL-13 induced the phosphorylation of signal transducers and activators of transcription 6 (STAT6) and increased M2 polarization in the radioresistance of BC [48]. Contrary to this, Fu et al. found that STAT6 increased myeloid cells’ polarization to M2 by the upregulation of IL-4 in lung cancer [49]. Similarly, Xue et al. used chlorogenic acid (CHA) to promote STAT1 activation, while inhibiting the activation of STAT6, subsequently, suppressing the polarization of M2 macrophages, and, as a result, inhibiting the tumor growth of glioblastoma in vivo [50]. Indeed, TAM polarization has been demonstrated to be modulated by the STAT6 signal cascade [51]. In sum, this evidence suggests that the relationship between IL-4 and STAT6 might be bidirectional, and any targeted strategy based on targeting STAT6 and IL-4 to block TAM polarization should be aware of their interaction in order to optimize therapeutic efficacy.

Additionally, extracellular proteins, such as endothelial growth factor (EGF), can induce M2 polarization by the activation of the EGF/PI3K/Akt/mTOR signaling pathway, and the EGFR antibody mAb225 and PI3K inhibitor LY294002 have been shown to suppress M2 polarization from monocytes in colon cancer [52,53]. However, recently, few studies have shown the potential targets of PI3K in TAM polarization. One of the reasons for this is that the activation of the oncogenic PI3K pathway is achieved in diverse ways, and inhibitors, such as LY294002, have shown limited therapeutic efficacy in preclinical trials [54,55]. In addition to those extracellular factors, the proteins expressed on the surface of cell membranes, such as S100A9, have been reported to be involved in TAM polarization [56]. Mechanistically, *Fusobacterium nucleatum*, a type of Gram-negative oral commensal anaerobe, has been found to upregulate S100A9 in macrophages and then promote M2 polarization through the TLR4/NF-κB signaling cascade [56]. Similarly, Kwak et al. found that MDSC-derived macrophages could express the S100A9 protein persistently, and S100A9 also promoted M2 polarization in metastatic melanoma [57]. Although the S100A9 inhibitor paquinimod has been extensively studied in inflammation [58,59], it is unknown if S100A9 inhibitors, such as paquinimod, are effective in TAM polarization, as no such data exist for cancers to date.

Lactate has worked as a direct regulator in TAM polarization [60]. Chen et al. found that tumor-cell-derived lactate-induced M2 polarization of TAMs can be mediated by the G-protein-coupled receptor 132 (Gpr132) in mouse models of BC [61], while no special antibody-targeted Gpr132 protein has been developed for suppressing TAM polarization. Further, Colegio et al. demonstrated that tumor-derived lactic acid promotes M2-like protumoral macrophages through HIF-1α stabilization [60]; conversely, Liu et al. and Tannahill et al. showed that glutamine-derived succinate promotes M1-like antitumoral macrophages through HIF-1α stabilization [62,63]. Based on these last findings, the reduction in succinate levels and the increasing α-ketoglutarate (α-KG)/succinate ratio by the blockade of the glutamine anaplerosis and gamma-aminobutyric acid (GABA) shunt pathway might inhibit TAM polarization in the antitumoral phenotype. Fatty acid oxidation (FAO) provides considerable energy for supporting macrophage polarization towards the M2 phenotype [64]. FAO is transcriptionally induced by peroxisome proliferation-activated receptor-gamma (PPARγ) [65,66]. However, Niu et al. showed that PPARγ plays a negative role during the pro-tumorigenic polarization of TAMs in BC models, and the caspase-1 inhibitor YVAD, which inhibit the caspase-1 mediated cleavage of PPARγ, attenuated the expression of markers specific for TAM polarization [65,67]. Furthermore, the activation of PPARγ is also attributed to a RIPK3 deficiency in TAMs. The lack of RIPK3 reduces ROS and significantly inhibits the caspase1-mediated cleavage of PPAR [68]. This indicates the paradoxical role of PPARγ in tumor progression, and such a discrepancy might be tissue specific or be controlled by unknown mechanisms in the downstream of the FAO metabolism. That M1 macrophages produce energy mainly through glycolysis, while M2 exhibits with a lower dependence on glycolysis and the TCA cycle occupies as the main source of ATP in M2 macrophages [69,70] indicates that glucose metabolism has a promising potential to be targeted for the conversion from M2 to M1 phenotype. To support it, Wei et al. performed the mannose-modified macrophage-derived microparticles (Man-MPs) loading metformin (Met@Man-MPs, a kind of intervener in the glucose metabolism) to target the M2-like macrophages, subsequently converting TAMs from the M2 to M1 phenotype [71]. Taken together, targeting metabolic methods for the inhibition of TAM polarization or the conversion the TAMs from the M2 to M1 phenotype is a promising approach in anti-tumor therapy.

## 3. Targeting Proteins in the Crosstalk between TAMs and Cancer Cells

**Figure 2:**
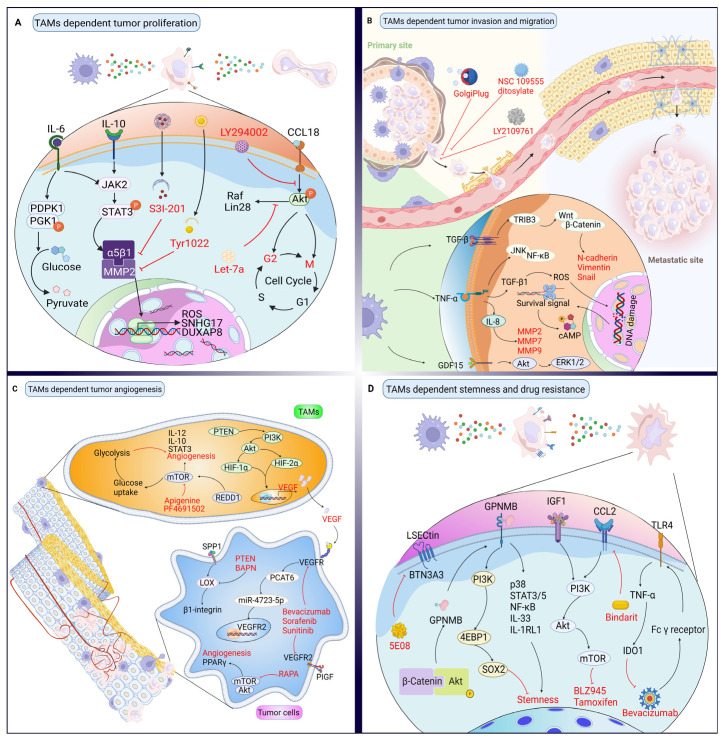
Targeting proteins in the crosstalk between TAMs and tumor cells. (**A**) In tumor cells, 3-phosphoinositide-dependent protein kinase 1 (PDPK1) is activated by TAMs derived IL-6, then phosphorylates phosphoglycerate kinase 1 (PGK1) to promote proliferation by pyruvate generation. TAM-derived IL-6 and IL-10 activate the JAK2/STAT3 cascade, then upregulate reactive oxygen species (ROS), SNHG17 and DUXAP8, to promote proliferation, while the S3I-201 and Tyr1022 could inhibit α5β1 integrin and MMP2, respectively, to inhibit this process. Similarly, TAM-derived CCL18 could activate the Akt signal, then prolong the S phase and reduce the G1 phase of cell cycle, and promote the expression of Raf and Lin28. LY294002 and Let-7a could inhibit the activity of the Akt cascade. (**B**) Grow factors and cytokines, such as transforming growth factor-beta (TGF-β), combine with their reporters in tumor cells, then activate the downstream effector, including cAMP and ROS, to promote the invasion and metastasis of tumor cells. The application of inhibitors, such as LY2109761, could block the invasion and metastasis of tumor cells. (**C**) Angiogenesis is regulated by several major signals, such as the PTEN/PI3K/Akt and mammalian target of rapamycin (mTOR) signaling pathways, and the TAM-derived vascular endothelial growth factor (VEGF) is also involved in this process. Bevacizumab, sorafenib, and sunitinib can neutralize VEGFR and VEGFR2 in tumor cells. The PTEN and beta-aminopropionitrile (BAPN) in tumor cells inhibit lysyl oxidase (LOX) dependent β1-integrin expression. Rapamycin (RAPA), Apigenine, and PF4691502 could block the mTOR signaling pathway to attenuate angiogenesis. (**D**) The cancer stemness would be mediated by LSECtin and the activity of β-Catenin/Akt pathway, while the β-Catenin/Akt pathway exhibits with a paradoxical role in the stemness of tumor cells. The IGF1 and CCL2 could bind with their receptors on tumor cells and then activate the PI3K/Akt/mTOR pathway to acquire the resistance of BLZ945 and Tamoxifen. Moreover, the metabolite of bevacizumab could interact with the Fc γ receptor to induce the production of TNF-α and indoleamine 2,3-dioxygenase 1 (IDO1) by interacting with TLR4 to acquire the resistance of bevacizumab. The bindarit would inhibit CCL2 to attenuate the generation of drug resistance.

Generally, plenty of clinical and experimental studies have suggested that tumorigenesis is promoted in a macrophage-dependent manner [4,72,73]. TAMs, which abundantly surround most solid tumors, could facilitate tumor progression through stimulating tumor proliferation, invasion, angiogenesis, and stemness, or by providing a physical barrier that attenuates anti-tumor immune responses (Figure 2). TAM-mediated tumor progression has been found to be highly dependent on the activity of various pathways and the phosphorylation of diverse proteins (Table 2). Thus, to suppress tumor progression, a number of studies have extensively focused on protein-targeted therapies in inhibiting the crosstalk between TAMs and tumor cells.

### 3.1. Effects of TAMs on Cancer Cell Proliferation and Their Targeted Therapy Based on Proteins

In addition to inducing TAM polarization, interleukin families, including IL-6 and IL-10, could promote cancer cell proliferation directly. For instance, in glioblastoma multiforme (GBM), Zhang et al. found that macrophage-derived IL-6 induced the phosphorylation of threonines (T243) in phosphoglycerate kinase 1 (PGK1), which was integral in GBM proliferation and the application of an anti-IL-6 antibody could abrogate this efficacy [74]. By Western blotting data, Mano et al. showed that TAM-derived IL-6 can promote HCC cell proliferation by inducing STAT3 phosphorylation and S3I-201 (a STAT3 inhibitor) could decrease IL-6-induced STAT3 phosphorylation for inhibiting HCC cell proliferation [75]. IL-6/STAT3 signaling has been found to be able to be mediated by integrin, a kind of membrane-spanning protein, and Kesanakurti et al. also provided findings illustrating that α5β1 integrin regulated IL-6/STAT3 signaling via interacting with matrix metallopeptidase 2 (MMP2) and Tyr1022, an α5β1 integrin inhibitor, can downregulate the activation of IL-6/STAT3 signaling and attenuate the proliferation of glioma [76], However, the targeting of the integrins of TAMs to inhibit tumor cell proliferation remains largely unexplored. Indeed, STAT3 can be activated by not only IL-6, but also IL-10. For instance, Yuan et al. reported that M2 macrophage secreted IL-10 could promote the proliferation of intrahepatic cholangiocarcinoma (ICC) cells through the STAT3 signaling pathway [77]. In sum, these studies suggest that TAM-associated tumor cell proliferation occurs in an IL-6 or IL-10/STAT3 signal cascade dependent manner and targeting proteins in the IL-6 or IL-10/STAT3 signaling pathway to attenuate tumor cell proliferation is promising in anti-tumor therapies.

Small-size secreted proteins, such as chemokines, are also involved in TAM-dependent tumor-cell proliferation. For instance, Wang et al. showed that M2 macrophage expressing CCL18 was associated with the activation of the FAK/PI3K/AKT pathway to promote the proliferation of esophageal squamous cell carcinoma (ESCC) cells, and that inhibiting PI3K by LY294002 to block the above pathway could impede the M2-induced proliferation of ESCC cells [78]. In another study, Wang et al. found that TAM-derived CCL18 could interfere with the cell cycle of BC cells by prolonging the S phase and reducing the G1 phase [79]. In detail, they used Let-7a to attenuate TAM-derived CCL18 induced proliferation of BC cells by downregulating the Lin28 and Raf-protein expression to prolong the G2/M phase and reduce the S phase [79]. By contrast, in T-cell lymphoma (CTCL), Günther et al. found that CCL18, which is derived from CD209^+^ macrophages in mycosis fungoides (MF), the most frequent form of cutaneous T-cell lymphoma (CTCL), did not induce proliferation in CTCL cell lines, including Hut78, SeAx, and MyLa; rather, CCL18 was found to inhibit the proliferation of both SeAx and MyLa [80]. These data suggest that the biochemical function of CCL18 for proliferation in solid tumors and hematoma might be divergent or tumor/tissue specific. Although its underlying mechanisms are still unclear, future anti-tumor treatment should be informed by the contradictory role of CCL18 in tumor progression in order to obtain good therapeutic efficacy.

### 3.2. Effects of TAMs on Cancer Cell Invasion and Their Targeted Therapy Based on Proteins

Tumor invasion, an integral step in distant metastasis, is typically induced by proteins dependent on the crosstalk between TAMs and tumor cells. For instance, Liu et al. found that TAM-derived transforming growth factor-β (TGF-β) could upregulate HIF-1α to increase the tribbles pseudokinase 3 (TRIB3) expression of colorectal cancer (CRC) cells, subsequently activating the β-catenin/Wnt signaling pathway, and as a result, facilitating the invasion of CRC cells [81]. Similarly, by transwell assay, Fan et al. showed that CD68^+^ TAMs mainly exhibited the M2 phenotype with a higher expression of TGF-β1, and could induce the EMT process and promote the invasive capability of HCC, while performing TGF-β1 neutralizing anti-body could attenuate TGF-β1 induced EMT, migration, and invasion of HCC [82]. Additionally, in a ESCC model, Okamoto et al. demonstrated that the TAM-derived growth differentiation factor 15 (GDF15) increased the phosphorylation of TGF-βRII in ESCC and promoted ESCC invasion, while LY2109761 (a TGF-βRI/II inhibitor) could suppress GDF15 dependent reinforcement of ESCC invasion [83]. Together, TGF-β and its super family-like GDF15 play a pivotal role in tumor invasion, and the EMT is regarded as an important mechanism in TGF-β induced tumor invasion.

Of note, a few targeting GDF15 inhibitors are available and further explorations are still needed to address the underlying mechanisms of GDF15 dependent on tumor invasion.

Further, tumor necrosis factor-α (TNF-α), a pro-inflammatory cytokine secreted by TAMs in the TME, has been found to support tumor invasion. Hagemann et al. observed that the co-culture between macrophages and ovarian or BC cells could induce the activation of the JNK and NF-κB pathways in a TNF-α-dependent manner, subsequently increasing the invasion of tumor cells; further, blocking the activation of JNK and NF-κB pathways by neutralizing antibodies can abrogate tumor cell invasiveness [84]. Additionally, Cho et al. found that M1 macrophages could secret TNF-α, and that the implementation of TNF-α inhibitor, TPCK, could suppress ovarian cancer invasion [85], indicating that M1/M2 macrophage-derived TNF-α promoting cancer invasion is a common event in tumor progression. To support this, in another study, Singh et al. found that macrophage-derived TNF-α induced the secretion of TGF-β1 in BC cells and then caused the DNA damage in BC cells by activating a survival pathway to deregulate DNA damage and ROS, subsequently leading to an increasing EMT by the upregulation of CREB phosphorylation and vimentin expression, while the neutralization of TNF-α by GolgiPlug (555029) could abrogate BC cell invasion and migration [86]. Interestingly, Watanabe et al. showed that exogenous recombinant TNF-α could induce the secretion of IL-8 in oral squamous cell carcinoma (OSCC) cells to increase OSCC cell invasion with the degradation of ECM via promoting the release of MMP2/7/9 [87]. In sum, the recombinant and TAM-derived TNF-α plays a promotive role in tumor invasion by various signaling pathways.

**Table 2:** Targeting proteins in the crosstalk between TAMs and cancer cells.

Ligand	Effector	Tumor	Inhibitor	Anti-Tumor Mechanism	Ref.
Inhibit the proliferation of cancer cells					
IL-10	PD-L1	NSCLC	BFD	Decrease IL-10 induced PD-L1 expression	[88]
IL-10	STAT3	RCC	N/A	Inhibit BMP-6 induced M2 polarization	[89]
MCAD	Lipid	BC	Sc-98926	Reduce LD accumulation in TAMs	[67]
MIF	IL-2	CRC	NIHIII.D.9	Decrease Treg generation and IL-2 production	[90]
EGFR	ILT4	NSCLC	Human ILT4 antibody	Inhibit TAM recruitment and M2 polarization	[91]
MK2	IL-1, IL-6, TNF-α	CRC	PF364402	Inhibit IL-1β, IL-6, and TNF-α, expression	[92]
Inhibit the invasion of tumor					
Lactate	Gpr132	BC	N/A	Inhibit lactate uptake and M2 macrophages activity	[61]
IGFBP2	FcγRIIB	GBM	Bs-1108R	Increase CD8+ T and p-CD19+ B cells and decreases M2 macrophages	[93]
S100A8/A9	MMP2, MMP9	LCC	N/A	Decrease MMP2 and MMP9	[94]
GS	Glutamine	N/A	MSO	Suppress M2 macrophages, induce T-cell recruitment	[95]
ATM	ATR	BC	Clone 10H11.E12	Decrease pCREB expression	[86]
Inhibit the angiogenesis of tumor					
IL-10/IL-13	N/A	RCC	Let-7d	Inhibit intratumoral macrophage M2 polarization	[47]
S100A7	JAB1	ESCC	N/A	Inhibit S1007A induced phosphorylation of ERK and FAK	[96]
N/A	PI3K/Akt/mTOR	HCC	Apigenin	Inhibit PI3K/Akt/mTOR pathway	[97]
S1PR1	NLRP3	BC	N/A	Inhibit S1PR1 dependent IL-1β expression	[98]
LOX	β1 integrin/PYK2	GBM	BAPN	Decrease TAM-derived SPP1	[99]
Inhibit the stemness of tumor					
α-KG	Jmjd-3	N/A	BPTES	Suppressed IL-4-induced STAT6 phosphorylation	[62]
LSECtin	BTN3A3	BC	5E08	N/A	[100]
CCL8	Erk1/2	GBM	SCH772984	Attenuate pseudopodia formation	[101]
IL-8	STAT3	OC	IL-8 Ab	Inhibit STAT3 and increase IL-12, NO	[102]
CBX8	H3K4me3	CRC	N/A	Increased the chemosensitivity of CRC cells	[103]

RCC: renal cell carcinoma; CRA: cervical cancer; ESCC: esophageal squamous cell carcinoma; EGF: endothelial growth factor; IGFBP2: insulin-like growth factor binding protein 2; Gpr132: G-protein-coupled receptor 132; PPARγ: peroxisome proliferation-activated receptor-γ; α-KG: α-ketoglutarate; GS: glutamine synthase; TNF-α: tumor necrosis factor-α; TGF-β: transforming growth factor-β; CHA: chlorogenic acid; MMP: matrix metalloproteinase; ERK: extracellular regulated protein kinase; mTOR: mechanistic target of rapamycin; MIF: macrophage migration inhibitory factor; MK2: MAPK-activated protein kinase 2; S1PR1: sphingosine-1-phosphate receptor 1; LOX: lysyl oxidase; LSECtin: liver sinusoidal endothelial cell lectin; CBX8: chromobox protein homolog 8; H3K4me3: histone H3 lysine 4 trimethylation; jmjd-3: Jumanji domain-containing protein D3; Gpr132: G-protein coupled receptor G2A; FcγRIIB: Fc gamma receptor IIB; PYK2: proline-rich tyrosine kinase 2; NLRP3: NOD-, LRR- and pyrin domain-containing protein 3; MSO: methionine sulfoximine; BPTES: bis-2-(5-phenylacetamido-1,3,4-thiadiazol-2-yl)ethyl sulfide; BAPN: beta-Aminopropionitrile monofumarate; BFD: bu fei decoction; MCAD: medium-chain acyl-CoA dehydrogenase; ATM: ataxia telangiectasia mutated; ATR attenuated total reflectance; N/A: not applicable.

### 3.3. Effects of TAMs on Angiogenesis and Their Targeted Therapy Based on Proteins

Tumor angiogenesis refers to abnormal blood vessel formation involving the migration, proliferation, and differentiation of endothelial cells, which is regulated by an extensive variety of angiogenic stimulators and inhibitors. It has been found that TAM-derived VEGF and its receptor are significant factors in promoting tumor angiogenesis. For instance, Joshi et al. provided evidence that revealed that the PTEN/PI3K/AKT signaling pathway could increase the hypoxia-induced HIF-1α and HIF-2α stability of macrophages to induce the secretion of VEGF for promoting tumor angiogenesis in Lewis lung carcinoma [104]. By the establishment of the BC model, Dong et al. showed that M2 macrophage derived VEGF could enhance the expression of prostate cancer-associated transcript 6 (PCAT6) and upregulate VEGFR-2 expression simultaneously by sponging miR-4723-5p, therefore reinforcing the tumor angiogenesis through the activation of VEGFR-2/Akt/mTOR signal axis [105]. In our preliminary study, we depleted macrophages in SCC models and found downregulated TGF-β1 and VEGFA in tumor cells [63], demonstrating that TGF-β1 reduction could be sufficient to reduce VEGFA-dependent angiogenesis after TAM ablation. Recently, the applications of VEGF and VEGFR inhibitors, including bevacizumab, sorafenib, and sunitinib, have been found to attenuate tumor angiogenesis by inducing endothelial cell apoptosis [106]. Tumor vasculature is surrounded by less smooth muscle cells, sinusoidal vessel plexuses and pericytes, missing lymphatic drainage and adrenergic innervation, erratically basal membrane, and interrupted endothelial lining, it also exhibits complex branching patterns and negatively influences drug delivery [107,108,109,110,111]; therefore, the exploring of the downstream pathway after VEGF stimulation might be a promising strategy for exploiting many more targets in order to overcome the above barriers in anti-tumor therapies.

Macrophage-derived secreted phosphoprotein 1 (SPP1) could also promote tumor angiogenesis in the phosphatase and tensin homolog (PTEN) deficiency GBM model [99]. In another study, Wenes et al. found that regulated in development and DNA damage 1 (REDD1), a negative regulator of mTOR, was upregulated in hypoxic TAMs, and that mTOR inhibition in TAMs could reduce an excessive angiogenic response, while blocking glycolysis by reducing glucose uptake, consequently inducing the formation of abnormal vascular [112]. To further explore the underlying mechanism, they provided evidence showing that REDD1 depletion could rescue the activation of mTOR and increase the glycolysis and/or glucose uptake of TAMs, subsequently competitively suppressing endothelial cells in a glucose-dependent manner and promoting the tumor vessel normalization [112]. Conversely, in HCC, Chen et al. reported that, when mTOR in macrophages was inhibited, STAT3 decreased the secretion of both IL-10 and IL-12 and could impede angiogenesis in vivo [113]. This suggests the dual role of mTOR in tumor angiogenesis; therefore, targeted mTOR strategies for attenuating tumor angiogenesis should be based on the further exploration of the underlying mechanism and the optimization of the anti-tumor function.

### 3.4. Effects of TAMs on Cancer Stemness and Drug Resistance, and Their Targeted Therapy Based on Proteins

Stemness, which confers proliferative ability on tumor cells, allows few tumor cells to develop and form visible tumors, and could promote tumorigenesis, therapeutic resistance, tumor recurrence, and dissemination [114,115]. TAM-mediated cancer stemness could be modulated by diverse proteins and related signaling pathways. For example, Liu et al. found that the LSECtin expressed on TAMs could interact with its receptor butyrophilin subfamily 3, member A3 (BTN3A3) and enhance the stemness of BC cells; further, when applying the anti-BTN3A3 antibody, 5E08, the stemness of BC cells could be attenuated [100]. Meanwhile, the activation of the Akt and β-catenin signaling pathway could induce macrophage-derived soluble glycoprotein NMB (GPNMB), which could bind with the CD44 receptor and then trigger crucial survival pathways, followed by increasing the IL-33, IL-1RL1 expression, p38, STAT3, STAT5 phosphorylation, and NF-κB activation, which would ultimately promote cancer stemness [116,117]. In our preliminary study, we found that targeting CD44 could decrease macrophage-associated PI3K/4EBP1/SOX2 signal for suppressing cancer stemness in head and neck cancer [118]. Similarly, when decreasing CD44 expression by the application of foretinib, the stemness of gastric cancer cells would be attenuated [119]. These data suggest that the activation of the β-catenin signaling pathway might be the key step in TAM-dependent tumor stemness, while both membrane proteins and soluble proteins are involved in this process. Other proteins, such as GPNMB and LSECtin, also exhibit potential to be used as targeted proteins in anti-tumor therapies.

Recently, new findings indicate that TAMs could mediate the therapeutic resistance of tumors in a protein-dependent manner. For instance, Li et al. provided evidence demonstrating that TAM-derived CCL2 could activate the PI3K/Akt/mTOR signaling pathway, subsequently promoting the acquirement of tamoxifenresistance in BC cells, and when applying Bindarit, a CCL2 synthesis inhibitor, the secretion of CCL2 and the phosphorylation of both Akt and mTOR could be significantly attenuated [120]. Additionally, in the CSF-1R inhibition therapy of GBM, Quail et al. found that the PI3K cascade can also be activated by TAM-derived insulin-like growth factors1 (IGF-1), as a result, promoting tumor cells to be resisted to BLZ945, a type of CSF-1R inhibitor, and the application of PI3K inhibitor (BKM120) could rescue the chemosensitivity of GBM in CSF-1R inhibition therapy [121]. These data suggest that PI3K might play a pivotal role in generating the drug resistance of tumors during therapies. Interestingly, the source of biomolecules that contribute to drug resistance is not always from TME; some side effects of the drugs in tumor therapies also induce drug resistance directly or indirectly. To support this, in the BC model, Liu et al. showed that bevacizumab-induced Fcγ receptor could interact with TLR4 and induce the M2b macrophages polarization; subsequently, the upregulation of indoleamine 2,3-dioxygenase 1 (IDO1) could be mediated in a TNF-α-dependent manner. Finally, the macrophage-derived TNF-α and IDO1 could induce drug resistance, and the neutralization of TNF-α could reverse bevacizumab resistance significantly [122]. Taken together, TAMs engage in crosstalk with tumor cells via diverse proteins and pathways to promote the proliferation, invasion, angiogenesis, stemness, and drug resistance of tumor cells, and these proteins hold great potential to be targeted in anti-tumor therapy (Table 3).

## 4. Targeting Proteins of TAMs in the Regulation of Tumor Immune Responses

**Figure 3:**
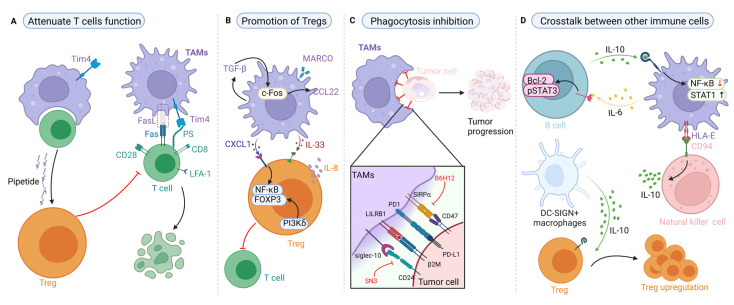
Targeting the crosstalk between TAMs and immune cells. (**A**) Tim4 and fasL expressed on the surface of TAMs could bind with phosphatidylserine and Fas on T cells, respectively, then inducing the apoptosis of T cells and suppressing the function of T cells. Apoptotic T cells could be phagocyted by TAMs, subsequently releasing pipetide to activate Treg, as a result, inhibiting the function of T cells. (**B**) TAM-derived chemokines and cytokines, such as TGF-β, CCL22, CXCL1, and IL-33, could upregulate the activity of Treg via the NF-κB and PI3Kδ signaling pathways, then suppressing the function of T cells. (**C**) The SIRPα, PD-1, LILRB1, and siglec-10 expressed on the surface of TAMs could bind with CD47, PD-L1, β2M, and CD24, respectively, then attenuating the phagocytosis of TAMs. Applying SN3 and B6H12 to block SIRPα/CD47 axis and siglec-10/CD24 axis, respectively, could reverse the inhibition of phagocytosis. (**D**) TAM crosstalk with other immune cells, such as B cells and natural killer cells, via the release of IL-6, IL-10, and the binding between HLA-3 and CD94. Even the DC-SIGN+ macrophages could express a high level of IL-10 to upregulate Tregs. BCL2: B-cell lymphoma 2; LFA-1: lymphocyte function-associated antigen-1; MARCO: macrophage receptor with collagenous structure; c-Fos: a proto-oncogene regulating the transcription of many genes.

TAMs serve an integral role in the alternation of the TME immune landscape and the establishment of anti-tumor immunity suppression. TAMs could engage in crosstalk with multiple immune cells, including CD8^+^ T cells, B cells, Tregs, dendritic cells (DCs), and natural killer (NK) cells, in a protein- and/or signaling-pathway-dependent manner (Figure 3). Therefore, to inhibit the immunosuppressive functions of TAMs, an extensive variety of studies have focused on targeting the effective proteins and pathways in the crosstalk between TAMs and immune cells.

### 4.1. Effects of TAMs on T-Cell Immunity and Their Targeted Therapy Based on Proteins

TAMs, especially M2 macrophages, are characterized by an impaired antigen presentation ability in the TME, the expression of associated proteins, the upregulation of immunosuppressive mediators, and the downregulation of proinflammatory cytokines; as a result, they can attenuate T-cell-mediated adaptive immune response [150,151]. Moreover, Miller et al. showed that Fas expressed on CD8^+^ T cells, binding with the FasL in macrophages, could activate intratumoral macrophages via the interferon-gamma (IFN-γ) and Fas/FasL axis, leading to a limitation of the immunosurveillance of intraocular tumors [152]. However, in another study on colorectal cancer with liver metastases, Yu et al. found that the hepatic CD11b^+^/F4/80^+^ macrophages express FasL highly when bearing liver tumors and tumor-specific T cells were siphoned into the liver in an integrin-dependent manner, subsequently leading to the activation of the extrinsic apoptosis of Fas^+^ T cells by the Fas–FasL pattern for promoting tumor progression [153]. This suggests the paradoxical role of the Fas/FasL axis in tumor-associated immune and relevant targeting therapies; the biochemical function of the Fas/FasL axis might be tissue specific. An alternative hypothesis, which is supported by the observation that studies on colorectal cancer without liver metastases have not reported altered T-cell numbers [153], is that liver metastasis might mediate immune function by the Fas/FasL axis, which prevents systemic anti-tumor immunity, and this effect should be recognized in anti-tumor therapies, especially for cancers with or without liver metastasis.

Another special protein expressed on the TAMs’ membrane is Tim4. For instance, Chow et al. found that TAMs in ovarian carcinoma can be divided into two subsets, including Tim4^+^ TAMs (F4/80^high^MHC-II^low^) and Tim4^-^ TAMs (F4/80^low^MHC-II^high^) [10]. Further, they suggested that Tim4^+^ on TAMs could combine directly with the phosphatidylserine in CD8^+^ T cells, subsequently attenuating its cytotoxicity and proliferation for promoting metastasis [10,154]. In another ovarian model, Xia et al. found that the immunosuppressive function of Tim4^+^ TAMs was mainly mediated by autophagy and the FAK family-interacting protein of 200 kDa (FIP200); further, they found that a deficiency in FIP200 could both result in Tim4^+^ TAMs’ death in the TME and promote T-cell-mediated anti-tumor immune response via the upregulation of ROS [155]. Similar to these findings, in our preliminary data, we also found that TAM ablation inhibited tumor growth, and that clodronate treatment for TAM depletion could increase the number of CD8^+^ T cells in SCC tumors [45]. In summary, this evidence suggests that TAMs could affect T-cell function by the membrane proteins; however, only a few inhibitors, such as targeting Tim4, have been made available to date. One of the reasons for this is that TAMs are highly heterogeneous stromal cells and their membrane proteins, such as Tim4, not only express on TAMs, but also on dendritic cells [156], B cells [156] and fibroblasts [157]; thus, the discovery of the heterogeneity of TAMs has revealed a remarkably complex and diverse portrait.

### 4.2. Effects of TAMs on Regulatory T Cells (Tregs) and Their Targeted Therapy Based on Proteins

Tregs, a specialized subset of CD4^+^ T cells identified as CD3^+^/CD4^+^/CD25^+^/FoxP3^+^ cells, act to suppress the immune response in maintaining immune homeostasis and self-tolerance. TAMs engage in crosstalk with Tregs by an extensive variety of proteins and associated pathways (Figure 3B). For instance, Fleur et al. provided evidence that applying a neutralizing antibody to inhibit the macrophage receptor with a collagenous structure (MARCO) in TAMs could downregulate the activation of Tregs [158]. In another study, Li et al. found that the CXCL1 expressed by TAMs could be recognized by CXCR2 of peripheral naive CD4^+^ T cells, subsequently recruiting those cells into the TME and inducing their differentiation into Tregs via the NF-κB/FoxP3 pathway in BC [20]. Using aiduqing can inhibit Tregs activity induced by TAM-derived CXCL1 and partly reverse the immunosuppressive TME [20]. Importantly, Wang et al. provided data that demonstrated that autocrine TGF-β stimulates TAMs to secret CCL22 via c-Fos to promote the recruitment of Tregs, and that the anti-CCL22 antibody could attenuate the recruitment of Tregs [138]. As feedback, the recruited Tregs could secrete IL-8 into the TME, and the exogenous IL-8 could induce the production of TGF-β in TAMs [138]. It suggests that there is positive feedback in the TAM-dependent recruitment of Tregs and that the establishment of the immunosuppressive TME might be not only induced by the biochemical function of TAMs, but also reinforced by the interaction between TAMs and immune cells, such as Tregs.

To further investigate the bilateral association between TAMs and Tregs, Gyori et al. showed that the depletion of either CSF1R^+^ TAMs or PI3Kδ-driven Foxp3^+^ Tregs in the immunosuppressive TME of colorectal cancer could induce the upregulation of the other and limit the therapeutic effect [159], suggesting that TAMs and Tregs serve as a couple of compensatory factors and maintain number homeostasis. Furthermore, after being recruited into the TME, Tregs could suppress the IFN-γ secretion of CD8^+^ T cells and attenuate the inhibition of sterol regulatory element-binding protein 1 (SREBP1) in immunosuppressive M2 macrophages, subsequently facilitating fatty acid synthesis in M2 macrophages [160], which indicates that Tregs maintain the metabolic fitness and survival of M2 macrophages indirectly. Of note, the SREBP1 inhibitor (Fatostatin) failed to elicit an effective anti-tumor response effectively [160], suggesting that the inhibition of SREBP1 might not affect Tregs, which have already been recruited into TME and permanently exert an immunosuppressive function. Taken together, Tregs serve as TAM-mediated immunosuppressive cells in TME, and the feedback between TAMs and Tregs, especially the IL-8/TGF-β axis, plays a pivotal role in the establishment of the immunosuppressive TME.

### 4.3. Effects of TAM-Mediated Phagocytosis and Their Targeted Therapy Based on Proteins

Macrophage-mediated phagocytosis, characterized by the uptake of macromolecules and larger particles through the membrane protrusions, can be triggered by diverse receptor–ligand interactions to clear pathogens, cellular fragments, and even dead cells for the initiation of innate immune response [161,162]. Interestingly, various signaling pathways and proteins in tumor cells could attenuate the innate immune function of TAMs (Figure 3C). For instance, Majeti et al. found that cluster of differentiation 47 (CD47), a kind of “do not eat me single”, expressed on the surface of tumor cells can protect tumor cells from phagocytosis by binding to signal regulatory protein alpha (SIRPα) in TAMs and that its inhibitor (B6H12.2) could enable the phagocytosis of acute myeloid leukemia cells in a mouse model [163]. Furthermore, Willingham et al. held that CD47 was a commonly expressed marker on all kinds of cancer cells, and that each human solid tumor cell requires CD47 to be expressed on the surface in order to evade phagocytic innate immune surveillance [164]. Of note, the inhibition of the CD47-SIRPα signaling pathway by rituximab alone in patients of follicular lymphoma led to anemia, and this side effect could be mitigated by 5F9 (a macrophage immune checkpoint inhibitor) [165,166], illustrating that combining rituximab for blocking CD47 and 5F9 for attenuating anemia could be an example of optimized antibody-dependent cellular phagocytosis. Since the swallowed tumor cells might be degraded by lysosomes in TAMs, to keep tumor cells alive, we previously hypothesized that TAMs phagocytize tumor cells via semi-phagocytosis to evade elimination by the immune system and avoid degradation by the endosomal/lysosomal system for distant metastasis [167].

Additionally, other proteins, including programed cell death receptor-1 (PD-1), leukocyte immunoglobulin-like receptor B1 (LILRB1), and CD24, expressed on the surface of TAMs and their receptors, could suppress TAM-mediated phagocytosis (Figure 3C). For instance, Gordon et al. found that the PD-1 expressed on the surface of TAMs could bind with the PD-L1, which exists on the membrane of colon carcinoma cells, subsequently attenuating the phagocytosis ability of TAMs [168], suggesting that the PD-1/PD-L1 axis not only suppresses the anti-tumor function of cytotoxic T cells, but also inhibits the phagocytosis of macrophages. In another study, Barkal et al. verified that cancer cells could express MHC class I component β2-microglobulin (β2M) on their surface and β2M could bind with leukocyte immunoglobulin-like receptor subfamily B member 1 (LILRB1), which exists on the surfaces of TAMs, subsequently protecting cancer cells from the phagocytosis of TAMs [169]. Specifically inhibiting the MHC class I/LILRB1 axis has been found to improve the phagocytosis of TAMs, subsequently exerting an anti-tumor effect [169,170]. Moreover, CD24 derived from tumor cells can interact with the inhibitory receptor sialic-acid-binding LG-like lectin 10 (siglec-10), which is highly expressed on the surface of TAMs, as a result, promoting the immune evasion and attenuating the phagocytosis of TAMs [171]. Inhibiting CD24 via clone SN3 increased the phagocytosis of TAMs and suppressed triple-negative BC progression [171]. In summary, the phagocytic inhibition of TAMs might be mediated by various proteins, and the targeting of one of them alone could exert a limited anti-tumor effect for the existence of alternative mechanisms, and combining with two or more kinds of drugs to target multiple proteins simultaneously might be an effective anti-tumor strategy.

### 4.4. Effects of TAMs on Other Immune Cells and Their Targeted Therapy Based on Proteins

Other immune cells, including B cells and NK cells, also engage in crosstalk with TAMs, and the proteins involved in the crosstalk have the potential to be targeted for anti-cancer therapy (Figure 3D). For instance, in a mouse model, Wong et al. provided evidence showing that IL-10 secreted by B cells promoted M2 polarization of TAMs via the downregulation of the NF-κB signaling pathway and the upregulation of the STAT1 signaling cascade in TAMs, subsequently promoting B16 melanoma progression [172]. Furthermore, human leukocyte antigen (HLA)-E expressed on the surface of TAMs could bind with CD94 on the membrane of NK cells to promote the release of IL-10 [173]. Although there is little evidence regarding the crosstalk between TAMs and DC cells, Liu et al. found that gastric cancer cells could induce TAMs to become DC-SIGN^+^ macrophages and express a high level of IL-10 in order to upregulate Tregs [174]. Together, this evidence suggests that IL-10 plays a pivotal role in the crosstalk between TAMs and immune cells during the tumor-associated immune response, and IL-10 maintains a potent target potential in anti-tumor therapies.

## 5. Conclusions and Future Perspectives

Currently, as summarized above, promising strategies targeting the proteins of TAMs have been developed in both in vitro and in vivo studies. Since TAMs play a paradoxical role in the TME, blocking TAM-maintained tumor promotion and taking advantage of their anti-tumor effect might be promising target strategies. Targeting TAMs in order to decrease the number of interleukins, including IL-4, IL-10, and IL-13, and the number of chemokines, such as CCL2 and CCL5, is a potential candidate for inhibiting TAM polarization and recruitment [175,176,177]. The selective elimination of tumor-promoting TAM subsets, such as M2 macrophages, or their repolarization from M2 to M1 to become anti-tumor elements might be effective therapeutic approaches. A direct anti-tumor strategy for targeting proteins in TAMs is blocking their crosstalk with tumor cells. For instance, IL-6 and IL-10 could promote cancer cell proliferation directly or indirectly [89,178] and TGF-β plays a pivotal role in tumor invasion [179]. Another strategy based on the receptor–ligand pattern aims at increasing immune responses through T cells, Tregs, and B cells, for instance. However, because of the highly heterogeneous nature of TAMs, the same or different types of proteins that are commonly targeted in TAMs and tumor cells might optimize the therapeutic efficacy and attenuate side effects. Macrophage-associated targeted approaches have already entered clinical practice [180]. Further works are also needed to explore the heterogeneity of TAMs in the TME, and they should aim at discovering novel immune cascades and nanomaterials based on the proteins of TAMs for therapeutic targets to produce superior antitumor effects and fewer side effects.

## Figures and Tables

**Table 1:** Targeting proteins for inhibiting TAM recruitment and polarization.

Target	Inhibitor	Tumor	Study Design	Anti-Tumor Mechanism	Ref.
Inhibit recruitment of TAMs					
CCL2	Celecoxib	GBM	C57BL/6 J mice + Eagle medium F-12	Decrease pNF-κB expression	[9]
	6-Shogaol	BC	MDA-MB-231/A549/4T1 cell line + Leibovitz’s L-15, F-12K, etc. medium	Decrease CCL2 by inhibiting STAT3 activation	[18]
CCR2	Losartan	BC	4T1-Luc, etc. cell line + ICR, etc. mice	Inhibit CCL2-induced p-ERK1/2	[19]
CXCL1	Aiduqing	BC	4T1/293 T cell line + BALB/c mice + DMEM/RPMI-1640	Decrease Tregs differentiation and infiltration	[20]
CCR5	Maraviroc	BC	MDA-MB-436/4T1.2 cell line + DMEM	Inhibit TAM recruitment	[21]
CCL5	HuR	BC	MCF-7/MDA-MB-231 cell line + DMEM	Inhibit CCL5 expression	[22]
CCR1	J113863	FA	NCTC 2472 cell line + NCTC 135 medium + C57BL/6, C3H/He mice	Inhibit thermal hyperalgesia	[23]
CXCR7	CCX771	BC	4T1 cell line + DMEM + BALB/c mice	Reduce p-STAT3 activation	[24]
CXCL8	IFN-γ	PC	BxPC-3, etc. cell line + C57BL/6 mice	Inhibit macrophages traffic	[25]
	ACPP Antibody	NPC	C666-1 cell line + RPMI 1640 medium	Inhibit PI3K/AKT pathway	[26]
IL-1β	Anakinra	BC	4T1 cell line + α-MEM + BALB/c mice	Inhibit CCL5, CXCX12 expression	[27]
IL-6	Siltuximab	OC	Tissue from ovarian cancer patients + endotoxin-free RPMI/DMEM medium	Reduce cytokine and chemokine, inhibit IL-6 signaling	[28]
S100B	Duloxetine	GLA	GL261-Luc/KR158B cell line + DMEM + *CX_3_CR_1_^GFP^* mice	Decrease CCL2 expression	[29]
CSF-1R	PLX3397	HCC	Hep3B/HepG2/THP-1, etc. cell line + OPN knockout C57BL/6 mice	Inhibit PPARγ activity to reduce TAM numbers	[30]
A2A	SCH58261	HCC	Tissue from HCC patients	Reduce Akt and ERK phosphorylation to reduce TAM numbers	[31]
MEK	GDC-0623	PC	PDA30364 cell line + pan monocyte isolation kit	Exterminate M2 macrophages	[32]
Inhibit the polarization of TAMs					
STAT6	Gefitinib	LLC	Cells from Chinese Academy of Sciences + DMEM + C57BL/6 mice	Inhibit IL-13/STAT6 pathway	[33]
CSF-1R	BLZ945	GLA	U-87 MG, etc. cell line + RCAS-hPDGF-B/Nestin-Tv-a; Ink4a/Arf^−/−^ mice	Inhibit heterotypic signaling	[34]
YAP	Ovatodiolide	CRC	HT-29, etc. cell line + Serum-Free Medium + NOD, SCID, BALB/c mice	Suppress IL-6 induced pathway	[35]
IL-6R	CPEB3	CRC	SW480/HCT116/LoVo, etc. cell line + BALB/c mice	Inhibit epithelial-mesenchymal transition	[36]
Ang-2	AS16	SA	Plasmid pPIC3.5K + BMMY + SD rat	Inhibit M2 polarization	[37]
Integrin β3	Sc-7312	BC	4T1/HEK293T cell line + RPMI-1640 and DMEM + BALB/c mice	Inhibit integrin β3 induced PPARγ activity	[38]
EP4	TP-16	CRC	CT26/4T1/HCT116 cell line + DMEM and F12 medium + C57BL/6, etc. mice	Reprogram IMCs, enhance tumor elimination	[39]
CD206	RP-182	PC	CD206^high^ M2-like macrophages + KPC, KP16 mice	Reduce M2-like TAMs, improve antitumor immune responses	[40]
PlGF	HRG	BT	T241/Panc02 cell line + C57BL/6, BALB/c mice	Promote vessel normalization, improve tumor perfusion	[41]

GLA: glioma; GBM: glioblastoma multiforme; HCC: hepatocellular carcinoma; PC: prostate cancer; CRC: colorectal cancer; PDAC: pancreatic ductal adenocarcinoma; BC: breast cancer; BT: breast tumor; BPT: breast phyllodes tumors; SC: squamous cancer; LC: lung cancer; LCC: Lewis lung cancer; SCLC: small cell lung cancer; NSCLC: non-small cell lung cancer; NPC: nasopharyngeal carcinoma tumor; FA: fibrosarcoma; SA: sarcoma; OC: ovarian cancer; CCL2: C–C motif chemokine 2; CXCL1: C–X–C motif chemokine 1; CXCR1: C–X–C motif chemokine 1; CCR2: C–C motif chemokine 2; IL-1β: interleukin 1 β: CSF-1R: colony-stimulating factor 1 receptor; YAP: Yes-associated protein; AMPK: AMP-activated protein kinase; Ang2: angiopoietin-2; MEK: MAPK/extracellular signal-regulated kinase; EP4: prostaglandin E2 (PGE2) receptor 4; HuR: human antigen R; CPEB3: Cytoplasmic polyadenylation element binding protein 3; AS16: 16-kilodalton protein: HRG: histidine-rich glycoprotein; STAT: signal transducer and activator of transcription.

**Table 3:** TAM-associated factors and their targeted roles in cancers.

Factor	Cancer	Recipient	Influence on Tumor	Biochemical Mechanism	Ref.
Cytokines					
IL-1β	HCC	Tumor	Promote tumor migration	NLRP3 dependent FAO/ROS/IL-1β axis	[123]
IL-6	CRC	Tumor	Promote tumor invasion and migration	Regulate JAK2/STAT3/miR-506-3p/FoxQ1 axis	[124]
	CRC	Tumor	Promote tumor invasion and migration	Activate the Wnt/β-catenin pathway	[125]
	BC	TAMs	Promote tumor development	Activate the gp130/STAT3 pathway	[126]
	HCC	Tumor	Promote tumor invasion and metastasis	Activate IL-6/ERK and STAT3 pathway	[127]
IL-8	OC	Tumor	Promote tumor stemness	Activate the IL-8/STAT3 pathway	[102]
IL-10	PC	Tumor	Promote tumor migration	Activate TLR4/IL-10 to express MMP2 and MMP9	[128]
	NSCLC	Tumor	Promote tumor invasion	Induce PD-L1 expression	[88]
	BC	DC	Attenuate CD8^+^ T-cell cytotoxicity	Decrease IL-12 expression	[129]
IL-23	KC	Treg	Promote tumor immune evasion	Increase IL-10, TGF-β expression, and Treg activity	[130]
	BC	TAMs	Promote tumor angiogenesis	Increase IL-10, TGF-β, VEGF expression	[131]
IL-34	CRC	TAMs	Promote tumor growth	Increase IL-6 expression	[132]
Chemokines					
CCL2	BC	Tumor	Promote drug resistance	Activate the PI3K/Akt/mTOR pathway	[120]
CCL5	CRC	Tumor	Promote tumor immune escape	Activate the p65/STAT3-CSN5-PD-L1 pathway	[133]
CCL8	GBM	Tumor	Promote tumor invasion and stemness	Activate the ERK1/2 pathway	[101]
CXCL12	CRC	Tumor	Promote tumor angiogenesis	Activate the MK2 pathway	[134]
CCL18	BC	Tumor	Promote tumor invasion and metastasis	Activate the AnxA2/PI3K/Akt/GSK3β/Snail pathway	[135]
	BC	Tumor	Promote tumor metastasis	Activate the PKCδ/STAT3, NF-κB pathway	[136]
CCL20	CRC	Treg	Promote Treg recruitment	CCL20/CCR6 couple	[137]
CCL22	NSCLC	Treg	Promote Treg recruitment	Increase IL-8 expression	[138]
Others					
TNF-α	BC	Tumor	Promote tumor EMT and migration	Increase cAMP and CREB expression	[86]
TGF-β	CRC	TAMs	Promote tumor proliferation	Increase RGC-32, COX2 expression	[139]
LSECtin	BC	Tumor	Promote tumor stemness	N/A	[100]
MIF	CRC	N/A	Promote tumor growth	Increase Tregs generation	[90]
	PDAC	N/A	Promote tumor metastasis	Activate AKT, ERK, and express cyclin-D1, MMP2	[140]
Xist	BC	TAMs	Promote tumor proliferation	lncRNA-Xist/miR-101-3p/KLF6/C/EBPα axis	[141]
ROS	CRC	N/A	Promote tumor proliferation	Activate NF-κB, AP-1, EGR-1	[142]
MCP-1	CRC	Tumor	Promote tumor growth, invasion	Activate the MK2 pathway	[92]
BMP-6	PC	TAMs	Promote tumor angiogenesis and growth	Increase IL-1a expression through Smad1, NF-κB	[143]
GPR35	CRC	Tumor	Promote tumor angiogenesis and growth	Na/K-ATPase-dependent ion pumping	[144]
CD206	CRC	N/A	Attenuate CD8^+^ T-cell cytotoxicity	Inhibit CD45 phosphatase activity	[145]
Oct4	LC	TAMs	Promote tumor growth	Increase M-CSF expression	[146]
Chi3L1	BC	Tumor	Promote tumor metastasis	Activate the CHI3L1/IL-13Rα2/ERK/JNK axis	[147]
RACK1	OSCC	TAMs	Promote tumor development	Regulate NF-κB pathway	[148]
GPNMB	BC	Tumor	Promote tumor stemness	Increase IL-33, CD44 expression	[116]
S100A9	HCC	Tumor	Promote tumor stemness	Activate AGER/NF-κB axis	[149]

OSCC: oral squamous cell carcinoma; KC: kidney cancer; COX2: cyclooxygenase 2; CtsZ: cathepsin Z; FRβ: folate receptor-beta; LCN-2: lipocalin 2; Xist: X inactive-specific transcript; VEGFR: vascular endothelial growth factor receptor; ROS: reactive oxygen species; MCP-1: monocyte chemoattractant protein-1; BMP-6: bone morphogenetic protein 6; GPR35: G protein-coupled receptor 35; Oct4: octamer-binding transcription factor 4; CTHRC1: collagen triple helix repeats containing 1; Chi3L1: chitinase 3-like protein 1; CD206: mannose receptor; RACK1: receptor for activated C kinase 1; GPNMB: glycoprotein NMB; IL-37: interleukin 37; α_m_β_2_: α_m_β_2_ integrin; CREB: cAMP response-binding protein; N/A: not applicable.

## Data Availability

Not applicable.
